# The potential of psychological intervention to decrease the risk of anxiety and depression in pulmonary fibrosis

**DOI:** 10.1080/07853890.2026.2743870

**Published:** 2026-10-22

**Authors:** Eda Sara Persentili, Mireia Baiges, Marta Jiménez, Miguel Sampayo-Cordero, Yolanda Galea-Colon, Raquel Cuevas, Jaume Bordas-Martinez

**Affiliations:** a Psychology Department, Hospital General de Granollers, Granollers, Barcelona, Spain; b Respiratory Department, Hospital General de Granollers, Granollers, Barcelona, Spain; c Patient Experience Department, Hospital General de Granollers, Granollers, Barcelona, Spain; d Research and Statistics Unit, Hospital General de Granollers, Granollers, Barcelona, Spain; e Department of Medicine, Faculty of Medicine and Health Sciences, Universitat Internacional de Catalunya, Sant Cugat del Vallès, Barcelona, Spain

**Keywords:** Anxiety, depression, integrated care, interstitial lung disease, psychological intervention, pulmonary fibrosis

## Abstract

**Background:**

Emotional distress in pulmonary fibrosis remains under-recognised and poorly addressed. This study assessed changes in anxiety and depressive symptoms following an individualised psychological intervention.

**Methods:**

Single-centre, single-arm before-and-after observational study of an individualised psychological intervention delivered in routine ILD care, combining acceptance and commitment therapy, humanistic-existential techniques, and psychophysiological interventions. Anxiety and depressive symptoms were assessed with the Hospital Anxiety and Depression Scale (HADS) before and after the intervention. Patient- and caregiver-reported experience measures (PREMs) were collected. Response was analysed across characteristics defined before any analysis of outcome data (age, sex, disease severity [ILD-GAP], frailty [CFS], time since diagnosis, and baseline HADS).

**Results:**

Thirty patients were included (50.0% women; mean age 71.0 years). At baseline, 76.7% scored ≥8 on HADS-anxiety and 76.7% on HADS-depression, indicatingclinically relevant symptom risk. Six patients discontinued (lung transplantation, palliative care, or death; *n* = 2 each). Among 24 evaluable patients, mean HADS-anxiety decreased from 11.3 to 6.6 (mean difference 4.7; 97.5% CI 2.6–6.9; *p* < 0.001) and HADS-depression from 10.7 to 5.5 (mean difference 5.2; 97.5% CI 3.2–7.2; *p* < 0.001). HADS-anxiety score improved in 19 patients, was unchanged in 3 and worsened in 2; HADS-depression score improved in 20, was unchanged in 2 and worsened in 2. No patient worsened in HADS category. No differences in response were detected by age, sex, disease severity, frailtyor time since diagnosis. Patient- and caregiver-reported experience scores were high (totalscore 9.4 [SD 0.7] in patients and 9.5 [SD 0.7] in caregivers).

**Conclusion:**

In this uncontrolled study, an individualised psychological intervention was associated with reduced anxiety and depressive symptoms and a lower proportion of patients at risk of clinically relevant anxiety and depression in pulmonary fibrosis. These findings suggest the potential of psychological care within routine ILD follow-up and require confirmation in randomised controlled trials.

## KEY MESSAGES

An individualised psychological intervention delivered within a specialist ILD clinic was associated with reduced HADS-anxiety and HADS-depression scores and with a lower proportion of patients at risk of clinically relevant anxiety and depression, with no patient worsening in HADS category; the intervention was well accepted by patients and caregivers.

No differences in response were detected by disease severity, frailty, time since diagnosis, age or sex, suggesting that eligibility for psychological care should not be restricted by disease stage, functional impairment or time since diagnosis.

These findings are exploratory and require confirmation in larger multicentre randomised trials before informing clinical practice.

## Introduction

Interstitial lung diseases (ILDs) are a heterogeneous group of rare disorders characterised by inflammation and fibrosis of the lung parenchyma [1]. Among them, idiopathic pulmonary fibrosis (IPF) is the most prevalent and carries the worst prognosis, with a median survival of 3–5 years comparable to aggressive cancers such as pancreatic or lung cancer [2,3]. Other fibrotic ILDs may follow a progressive pulmonary fibrosis (PPF) phenotype with a clinical trajectory similar to IPF [3,4].

Pirfenidone and nintedanib slow forced vital capacity (FVC) decline and improve survival in IPF and PPF [5–7]. Nevertheless, these drugs are frequently associated with adverse effects, and patients continue to experience persistent dyspnoea, cough, and functional limitation [8–10]. Comprehensive care programmes address these needs [11–13], yet the psychological burden of living with a progressive, life-limiting respiratory disease remains a major unmet need and is rarely managed within routine respiratory care.

Anxiety and depression are highly prevalent among patients with pulmonary fibrosis, with reported rates ranging from 20% to over 60% [14–19]. These symptoms substantially impair health-related quality of life and may influence clinical outcomes [14,20]. International guidelines endorse psychological support as part of multidisciplinary care [3,21], but its implementation in routine clinical practice remains uncommon and poorly studied. As in other life-limiting illnesses, this distress is frequently sub-syndromal yet clinically significant, often goes unrecognised in routine respiratory care, and is amenable to intervention; models of integrated and collaborative psychological care developed in oncology have rarely been transposed to fibrotic ILD.

Despite the recognised burden of emotional distress in pulmonary fibrosis, evidence on psychological interventions remains scarce and largely limited to remote modalities [18,20,22], with inconsistent benefits in adjacent chronic respiratory diseases [23]. To our knowledge, no face-to-face, individualised, integrative psychological intervention has been evaluated in pulmonary fibrosis.

The main aim was to assess changes in anxiety and depressive symptoms following an individualised psychological intervention in patients with pulmonary fibrosis. Secondary aims were to describe (a) the consistency of the pre–post changes across predefined subgroups (age, sex, disease severity, frailty, time since diagnosis, and baseline anxiety and depressive symptoms); (b) the influence of additional patient and disease characteristics; and (c) patient- and caregiver-reported experience measures (PREMs).

## Methods

### Study design and participants

This was a single-centre, single-arm before-and-after observational study with prospective follow-up, evaluating an individualised psychological intervention delivered from 29 April 2024 as part of routine clinical care in a specialist ILD clinic. Psychological referral was offered by the treating pulmonologist and the ILD nurse specialist to patients with fibrotic ILD in whom emotional distress was clinically suspected at routine respiratory visits; no systematic screening was applied, and referral was not registered prospectively. Anxiety and depressive symptoms were assessed with the Hospital Anxiety and Depression Scale (HADS) by the psychologist at entry into psychological follow-up, and this assessment provided the baseline score. The study was approved by the institutional ethics committee on 25 March 2025 (CEIm: 20253005); for patients already attending the psychology clinic before that date, previously recorded assessments were retrieved from clinical records once consent had been obtained, and all subsequent assessments were collected prospectively. The last patient included in the analysis entered psychological follow-up on 22 July 2025, and data collection closed on 31 December 2025. HADS-anxiety and HADS-depression were designated as co-primary outcomes, and the subgroup analyses were defined before any analysis of the data was undertaken; a sample of 30 patients was planned (see Sample size section in the Supplementary Material). Post-intervention HADS and patient- and caregiver-reported experience measures were collected at discharge or at data-collection closure for patients still in active follow-up. This study is reported in accordance with the STROBE statement for observational studies.

### Eligibility criteria

Eligible patients had (a) an ILD diagnosis confirmed by a multidisciplinary committee [1,3]; and (b) the ability to attend psychological sessions in person or by telephone. Exclusion criteria were lack of informed consent, major psychiatric disorder, ongoing psychological treatment, or cognitive impairment. Patients were considered evaluable if they completed at least a baseline and a post-intervention HADS assessment. Because referral was offered on clinical grounds and therefore favoured patients with suspected distress, the study population corresponds to patients in whom emotional distress is clinically suspected rather than to an unselected fibrotic ILD population.

### Data collection

Demographic, clinical, and treatment variables were recorded, including ILD subtype, pulmonary function (FVC, diffusing capacity for carbon monoxide [DLCO]), dyspnoea (modified Medical Research Council scale [mMRC]), oxygen therapy, opioid use, and transplant status. Disease severity was assessed using the ILD-Gender, Age, Physiology (ILD-GAP) index [24] and the Clinical Frailty Scale (CFS) [25], which ranges from 1 (very fit) to 9 (terminally ill). Psychological variables included history of anxiety or depression in primary care records, predominant concerns, assigned by the psychologist to one of five pre-specified categories (loss of autonomy, disease progression or death, treatment tolerance, secondary losses, or other), bereavement, and support network availability. Psychotropic medication was retrieved from pharmacy dispensing records in all patients with paired assessments and classified as antidepressant or benzodiazepine/hypnotic, and as present at baseline or started, modified or withdrawn during follow-up.

Anxiety and depressive symptoms were assessed using the Hospital Anxiety and Depression Scale (HADS) [26], a self-reported screening instrument that quantifies symptom burden and identifies patients at risk of anxiety and depression rather than establishing a clinical diagnosis. Each subscale ranges from 0 to 21, and scores were categorised per subscale as 0–7 normal, 8–10 mild, 11–14 moderate, and 15–21 severe [26,27]. Continuous scores were reported as anxiety and depressive symptoms, and results based on the cut-off of 8 as risk of clinically relevant anxiety or depression. Assessments were performed at baseline and post-intervention. Patient- and caregiver-reported experience measures (PREMs) were collected post-intervention using an ad hoc 8-item questionnaire on a 0–10 scale, covering helpfulness, satisfaction, perceived improvement, and recommendation, and were analysed in the evaluable population.

At the initial psychological visit, the treating psychologist assigned each patient a single predominant coping style on the basis of the clinical interview, using the five domains of the Mini-MAC framework (fighting spirit, helplessness-hopelessness, anxious preoccupation, cognitive avoidance, and fatalism) as a conceptual reference [28]. The questionnaire was neither administered nor scored; this classification was exploratory rather than standardised. The Mini-MAC has not been psychometrically validated in fibrotic ILD.

### Outcomes

The primary outcome was the change in HADS-anxiety and HADS-depression subscale scores between baseline and post-intervention assessment. Secondary outcomes included PREM scores from patients and caregivers after the intervention. Patient characteristics were defined, before any analysis of outcome data was undertaken, for subgroup analyses of HADS change. These were age, sex, disease severity, frailty, time since diagnosis, and baseline HADS levels.

### Psychological intervention

The intervention used an integrative model combining humanistic, cognitive-behavioural, and psychophysiological approaches. Humanistic-existential work, including person-centred therapy to strengthen the therapeutic alliance and facilitate emotional expression within a meaning-centred framework [29], was undertaken when patients raised questions of meaning or existential concerns. Acceptance and commitment therapy (ACT), focusing on acceptance, cognitive defusion, and values-based action [30], was applied to support acceptance of the illness and the identification of goals that gave meaning to the illness experience. Cognitive restructuring, analysis of core beliefs, and psychophysiological techniques, namely diaphragmatic breathing and progressive muscle relaxation grounded in models of autonomic nervous system regulation [31], were applied when anxiety or dysregulation symptoms such as insomnia or rumination interfered with wellbeing, and when dysfunctional thoughts were identified. Components were selected according to the needs identified in each patient rather than delivered in a fixed sequence.

The intervention followed a common structure without a fixed protocol, comprising one to two initial sessions of joint assessment and goal-setting, intervention sessions, and a closing session. Apart from the HADS, assessment was based on clinical interview, without further structured questionnaires. All sessions were individual, lasted approximately one hour and were delivered by the same psychologist, who had more than 10 years’ experience in psycho-oncology and palliative care. Sessions were held in the outpatient psychology clinic, on days scheduled independently of respiratory visits and according to psychological need; the first session was always face to face and, in patients with difficulties travelling to the clinic, subsequent sessions, including the closing session, could be conducted by telephone. No standardised materials or home-based exercises were distributed, although individually tailored between-session tasks were agreed in some patients. Discharge was decided by mutual agreement between patient and psychologist, without any predefined score. Post-intervention HADS was administered at discharge, or after a minimum of three sessions in patients still in follow-up at closure. Caregivers did not receive psychological intervention themselves; their role was limited to providing caregiver-reported assessments of their relative’s experience. Cases were discussed with the referring pulmonologist and ILD nurse specialist whenever any of them considered it necessary.

### Statistical analysis

The primary analysis compared pre- and post-intervention HADS-anxiety and HADS-depression scores using paired data from patients with complete assessments. A two-sided type I error rate of 0.05 was allocated equally between the two primary outcomes, yielding a significance threshold of 0.025 per outcome. A sample size of 30 patients was planned (see Sample size section in the Supplementary Material).

Pre- and post-intervention HADS scores were analysed using the paired *t* test, with mean differences and their 97.5% confidence intervals reported. Normality of paired differences was assessed with the Shapiro–Wilk test. The Wilcoxon signed-rank test and Hodges–Lehmann location-shift estimator were applied as complementary non-parametric analyses.

A sensitivity analysis was conducted to assess the robustness of the results to potentially informative missingness due to intercurrent events (death, palliative home care, or transplantation), which are likely associated with clinical deterioration. Post-intervention HADS scores for patients with missing follow-up data were imputed with values ranging from their observed baseline score to the maximum possible score (21 points), and the *t* test was re-applied at each imputed value. The maximum deterioration from baseline immediately prior to the loss of statistical significance (*p* ≥ 0.025) was reported as the tipping point.

Patients were classified as improved, unchanged or worsened according to the direction of change in each HADS subscale. For the subgroup and regression analyses, response was instead defined by HADS category, as a shift to a less severe category between baseline and post-intervention assessment. The proportion achieving a minimal important difference of 1.7 points, the threshold used in the sample size calculation [32] and a value within the range of the IPF-specific estimates [33], was calculated among those with a baseline subscale score of 8 or above. Subgroup analyses were conducted to describe treatment response across patient subgroups. Subgroups were defined before any analysis of outcome data was undertaken, on the basis of clinical judgement, as those most likely to influence response to the psychological intervention. These analyses were exploratory and the study was not powered for them. Logistic regression models were used to estimate odds ratios and 95% confidence intervals. Multivariable logistic regression with repeated five-fold cross-validation was used to identify characteristics independently associated with improvement (see Supplementary Material). Patient and caregiver PREMs were compared using Spearman’s rank correlation coefficient. Associations between patient characteristics and changes in HADS scores from baseline to post-intervention (improved versus not improved) were also explored.

For secondary and exploratory analyses, a two-sided significance level of 0.05 was used, provided both primary outcomes were significant at the 0.025 threshold. No adjustment for multiple comparisons was applied to exploratory analyses, and their *p* values are considered descriptive. Further details are provided in the Statistical analysis section of the Supplementary Material.

## Results

### Patient disposition and intervention

Thirty patients were included. Six did not complete the post-intervention HADS assessment because of intercurrent clinical events unrelated to the intervention, namely lung transplantation (*n* = 2), transition to palliative care (*n* = 2) or death (*n* = 2). Twenty-four completed paired evaluations, and caregiver PREMs were available for 18 (Figure 1). Of the 30 patients, 25 (83.3%) were seen exclusively face to face and 5 (16.7%) had one or more sessions by telephone. Among the 24 evaluable patients, the median number of sessions was 6 [4;8], the median interval between sessions 63 days [54;71], and the median duration of the intervention period 10.9 months [6.0;16.0]. At closure, 13 of the 24 evaluable patients (54.2%) had been discharged after achieving the agreed objectives, after a median of 6 sessions [3;7], and 11 (45.8%) remained in active psychological follow-up.

**Figure 1.**
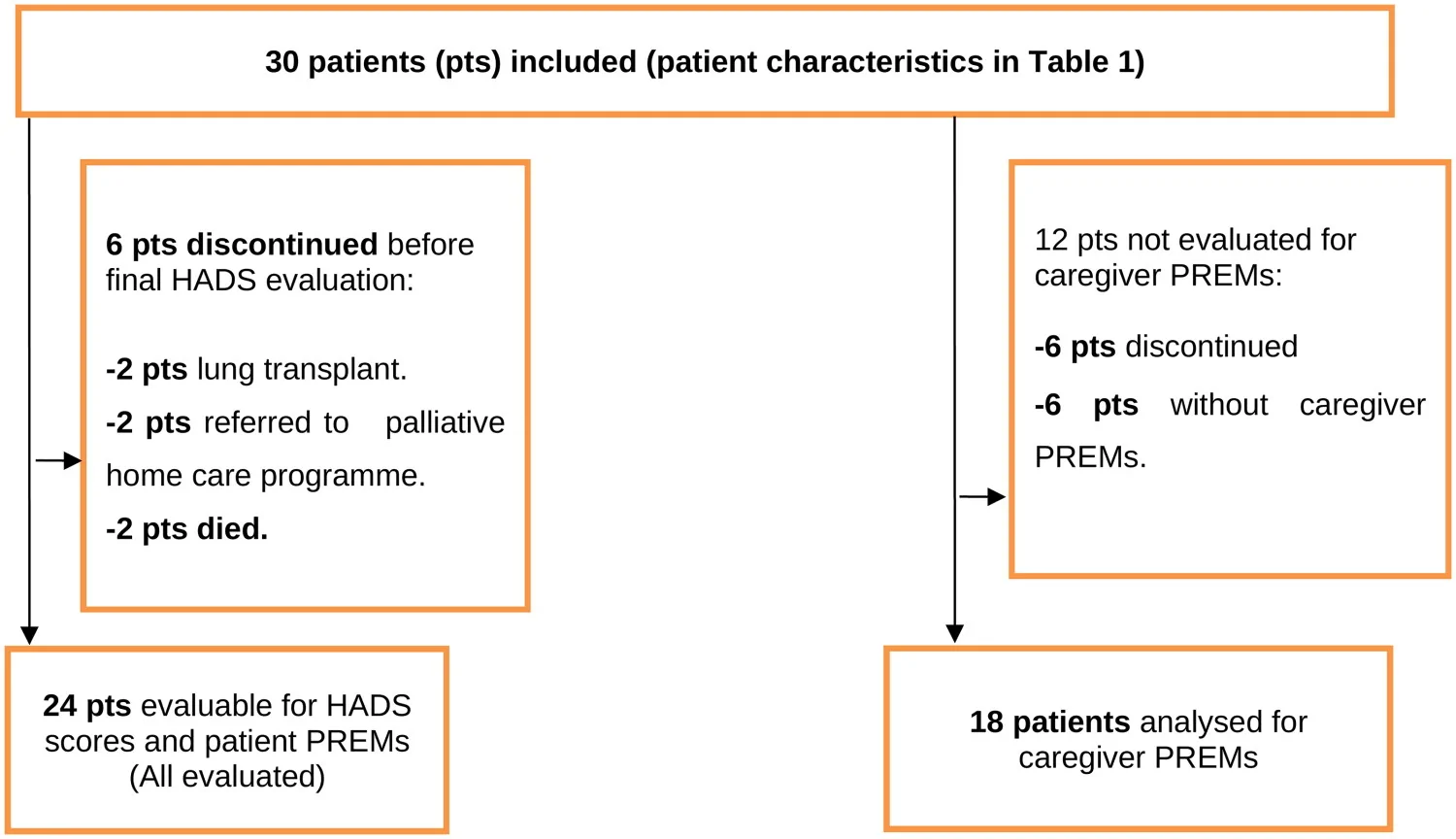
Study flowchart. **HADS**: Hospital Anxiety and Depression Scale. Six patients discontinued before post-intervention HADS evaluation (two lung transplantation, two referred to home palliative care, two deceased). Six additional caregivers did not complete the PREMs questionnaire.

### Baseline characteristics

Baseline characteristics are presented in Table 1. Mean age was 71.0 years (SD 8.3), and 50.0% were women. The most frequent diagnoses were chronic hypersensitivity pneumonitis (36.7%) and IPF (33.3%). Mean FVC was 72.4% predicted (SD 20.9) and DLCO 48.7% predicted (SD 15.4). An ILD-GAP index ≥4 was present in 56.7% and a CFS ≥5 in 46.7%. Antifibrotic drugs were used by 90.0%, supplemental oxygen on exertion by 43.3%, and opioids by 36.7%. Mean time since diagnosis was 18.7 months (SD 19.2), with 46.7% diagnosed within the preceding 12 months.

**Table 1.** Baseline clinical, social, and psychological characteristics of the patients.

Characteristics	Total *N* = 30
**Sociodemographic characteristics**	
Male, *n* (%)	15 (50.0%)
Age, mean (SD)	71.0 (8.3)
Civil status, *n* (%)	
Married	22 (73.3%)
Widowed	6 (20.0%)
Divorced	1 (3.3%)
Single	1 (3.3%)
Support network available, *n* (%)	29 (96.7%)
Effective support network, *n* (%)	24 (80.0%)
**Disease and clinical characteristics**	
ILD diagnosis, *n* (%)	
Chronic HP	11 (36.7%)
IPF	10 (33.3%)
CTD-ILD	4 (13.3%)
IPAF	3 (10.0%)
Other	2 (6.7%)
FVC (% of predicted), mean (SD)	72.4 (20.9)
DLCO (% of predicted), mean (SD)	48.7 (15.4)
ILD-GAP index ≥4, *n* (%)	17 (56.7%)
CFS ≥5, *n* (%)	14 (46.7%)
Time since diagnosis in months, mean (SD)	18.7 (19.2)
≤6 months, *n* (%)	8 (26.7%)
≤12 months, *n* (%)	14 (46.7%)
Dyspnoea (mMRC), n (%)	
Grade 1	11 (36.7%)
Grade 2	7 (23.3%)
Grade 3	12 (40.0%)
Family aggregation, *n* (%)	6 (20.0%)
**Treatment and supportive care**	
Antifibrotic therapy, *n* (%)	27 (90.0%)
Antifibrotic side effects, *n* (%)	4 (13.3%)
O2 on exertion, *n* (%)	13 (43.3%)
O2 at rest, *n* (%)	4 (13.3%)
Opioid use, *n* (%)	11 (36.7%)
Lung transplant evaluation, *n* (%)	6 (20.0%)
**Psychological history and baseline status**	
Anxiety and/or depression (primary care record)^a^, *n* (%)	7 (23.3%)
Fibromyalgia, *n* (%)	3 (10.0%)
Bereavement, *n* (%)	7 (23.3%)
Predominant concern, *n* (%)	
Loss of autonomy	12 (40.0%)
Disease progression or death	9 (30.0%)
Secondary losses	2 (6.7%)
Treatment tolerance	2 (6.7%)
Other	5 (16.7%)
Information integration, *n* (%)	
Full	23 (76.7%)
Partial	7 (23.3%)
Coping style at baseline^b^, *n* (%)	
Anxious preoccupation	9 (30.0%)
Fatalism	9 (30.0%)
Cognitive avoidance	6 (20.0%)
Helplessness-hopelessness	4 (13.3%)
Fighting spirit	2 (6.7%)
**HADS baseline**	
Anxiety subscale, mean (SD)	11.5 (4.5)
Anxiety level, *n* (%)	
Severe	10 (33.3%)
Moderate	7 (23.3%)
Mild	6 (20.0%)
Normal	7 (23.3%)
Depression subscale, mean (SD)	10.9 (4.4)
Depression level, *n* (%)	
Severe	5 (16.7%)
Moderate	11 (36.7%)
Mild	7 (23.3%)
Normal	7 (23.3%)

Values are *n* (%) or mean (standard deviation).

**ILD**: interstitial lung disease. **HP**: hypersensitivity pneumonitis. **CTD-ILD**: connective tissue disease-associated ILD. **IPAF**: interstitial pneumonia with autoimmune features. **FVC**: forced vital capacity (% predicted). **DLCO**: diffusing capacity for carbon monoxide (% predicted). **ILD-GAP**: Interstitial lung disease-Gender, age, Physiology index. **CFS**: Clinical Frailty Scale. **mMRC**: modified Medical Research Council dyspnoea scale. **HADS**: Hospital anxiety and Depression Scale. **SD**: standard deviation.

ᵃDocumented in primary care records without formal psychiatric or psychological evaluation.

^b^Clinically assigned by the treating psychologist; not psychometrically scored (see ‘Methods’).

At baseline, 76.7% scored ≥8 on HADS-anxiety, indicating risk of clinically relevant anxiety (33.3% severe, 23.3% moderate, 20.0% mild), and 76.7% scored ≥8 on HADS-depression (16.7% severe, 36.7% moderate, 23.3% mild). Mean HADS-anxiety was 11.5 (SD 4.5) and HADS-depression 10.9 (SD 4.4). Pre-existing anxiety and/or depression in primary care records was present in 7 patients (23.3%); none had undergone formal psychological evaluation. Among the 24 patients with paired assessments, 4 (16.7%) were receiving an antidepressant and 7 (29.2%) a benzodiazepine or hypnotic, none initiated by the ILD unit and none preceded by formal psychological assessment.

At baseline, the predominant coping style was classified as anxious preoccupation in 9 patients (30.0%), fatalism in 9 (30.0%), cognitive avoidance in 6 (20.0%), helplessness–hopelessness in 4 (13.3%) and fighting spirit in 2 (6.7%) (Table 1).

### Impact of the psychological intervention on anxiety and depressive symptoms

The intervention was associated with statistically significant reductions in both anxiety and depressive symptoms among the 24 evaluable patients (Figure 2). Mean HADS-anxiety decreased from 11.3 (SD 4.8) to 6.6 (SD 3.6), with a mean difference of 4.7 (97.5% CI 2.6–6.9; *p* < 0.001). Mean HADS-depression decreased from 10.7 (SD 4.6) to 5.5 (SD 3.4), with a mean difference of 5.2 (97.5% CI 3.2–7.2; *p* < 0.001). For HADS-anxiety score, 19 patients (79.2%) improved, 3 (12.5%) were unchanged and 2 (8.3%) worsened; for HADS-depression score, 20 (83.3%) improved, 2 (8.3%) were unchanged and 2 (8.3%) worsened. No patient worsened in HADS category on either subscale. Among patients with a baseline subscale score of 8 or above, 13 of 17 (76.5%) reached the minimal important difference of 1.7 points for anxiety and 16 of 18 (88.9%) for depression [32]. The proportion of patients with a severe HADS-anxiety score decreased from 33.3% to 0%, and with a severe HADS-depression score from 16.7% to 4.2%. The proportion scoring ≥8, indicating risk of clinically relevant symptoms, fell from 70.8% to 33.3% for anxiety and from 75.0% to 16.7% for depression (Figure 2). No adverse events related to the intervention were reported, and no patient discontinued sessions due to therapy-related distress. During the intervention period, one patient started lorazepam for insomnia and one had diazepam switched to trazodone, an antidepressant prescribed for insomnia; both were prescribed in primary care. No psychotropic medication was withdrawn, and no drug was started or modified for anxiety or depression.

**Figure 2.**
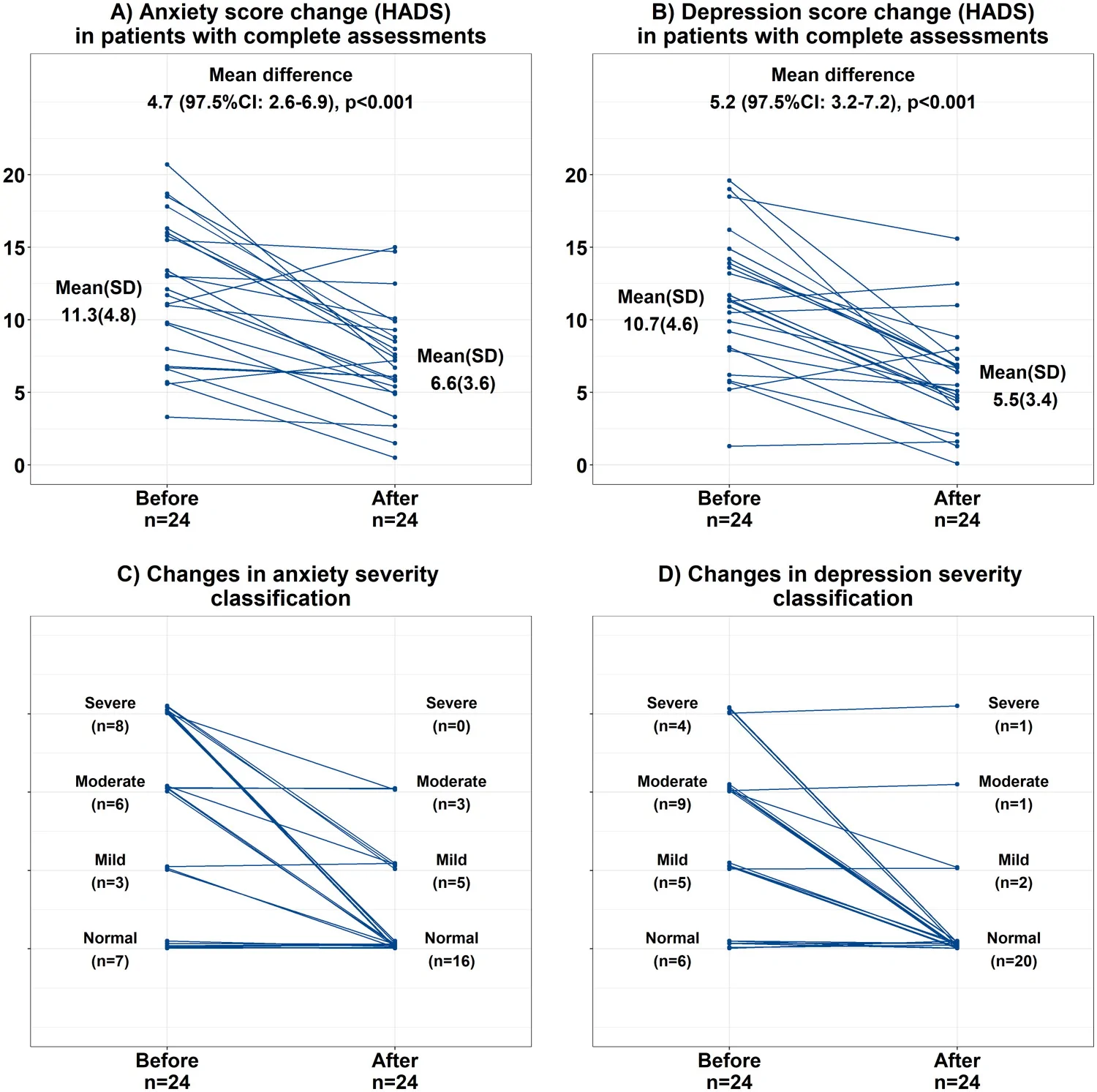
Hospital Anxiety and Depression Scale (HADS) anxiety and depression subscale scores before and after the psychological intervention in evaluable patients (*n* = 24). HADS score thresholds: 0–7 normal, 8–10 mild, 11–14 moderate, 15–21 severe. Individual patient trajectories are shown. Pre- and post-intervention changes in HADS scores were analysed using paired *t* test mean differences and associated 97.5% confidence intervals. The *p* values from the Shapiro–Wilk test for the differences in HADS-anxiety and HADS-depression scores were 0.686 and 0.466, respectively. The Wilcoxon signed-rank test yielded a Hodges–Lehmann location-shift estimate of 5.5 points for both the anxiety (97.5% CI: 3–8) and depression (97.5% CI: 3.5–7.5) subscales (both *p* < 0.001).

As a sensitivity analysis, the six patients who discontinued due to intercurrent events were assigned a post-intervention HADS score of 18 (Supplementary Figure 1), the tipping point value immediately prior to losing statistical significance (*p* ≥ 0.025). Mean HADS-anxiety decreased from 11.5 (SD 4.5) to 8.9 (SD 5.6), with a mean difference of 2.6 (97.5% CI 0.1–5.2; *p* = 0.022). Mean HADS-depression decreased from 10.9 (SD 4.4) to 8.0 (SD 5.9), with a mean difference of 2.9 (97.5% CI 0.6–5.2; *p* = 0.016).

### Response to the intervention according to predefined patient characteristics

No statistically significant differences in HADS response were detected across age, sex, disease severity, frailty or time since diagnosis, for either HADS subscale (Figure 3). Baseline HADS score was the only predefined characteristic associated with improvement in HADS category (anxiety: cross-validated AUC 94.7%, 95% CI 93.3–96.0%; depression: cross-validated AUC 83.8%, 95% CI 80.8–86.7%).

**Figure 3.**
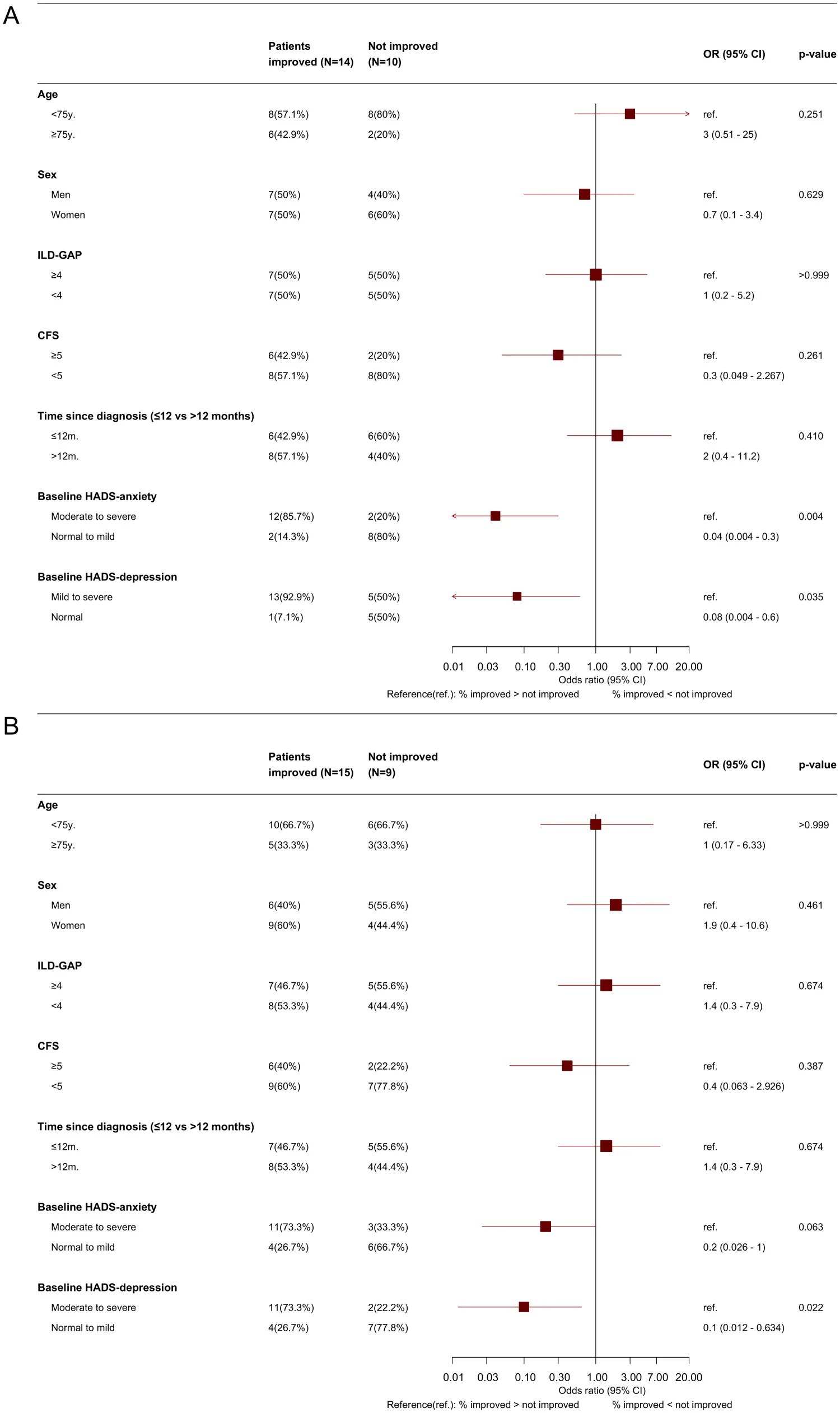
Improvement in HADS anxiety (A) and HADS depression (B) across predefined patient characteristics (forest plot). Improvement is defined as a shift to a less severe HADS category. Odds ratios and 95% confidence intervals for improvement in HADS category. An odds ratio lower than 1 indicates higher odds of improvement in the reference group compared with the non-reference group, whereas an odds ratio greater than 1 indicates lower odds of improvement. **ILD-GAP**: Interstitial Lung Disease-Gender, Age, Physiology index. **CFS**: Clinical Frailty Scale. HADS: Hospital Anxiety and Depression Scale. OR: odds ratio. CI: confidence interval. *p* values from Wald tests. A. The cross-validated area under the receiver operating characteristic curve (AUC) for baseline HADS anxiety was 94.7% (95% CI: 93.3–96.0%). In the multivariable model, baseline HADS anxiety was the only patient characteristic retained as an independent predictor of improvement. B. The cross-validated AUC for baseline HADS depression was 83.8% (95% CI: 80.8–86.7%). In the multivariable model, this was the only patient characteristic retained as an independent predictor.

### Response to the intervention according to other patient characteristics

In exploratory univariate analyses, bereavement was more frequent among patients whose HADS-depression category improved (7/15 vs 0/9; *p* = 0.022); however, this association was no longer observed after adjustment for baseline HADS-depression score (*p* = 0.996). No other characteristic was associated with response. Full comparisons are presented in Supplementary Table 1 for HADS-anxiety and Supplementary Table 2 for HADS-depression, including dyspnoea (mMRC; *p* = 0.497 and *p* = 0.488) and cough (*p* > 0.999 for both). Among the 24 evaluable patients, a support network was available in 95.8% and effective in 79.2%, with no association with response. Baseline HADS scores did not differ between patients with and without pre-existing anxiety and/or depression (*n* = 7 vs *n* = 23; HADS-anxiety: 12.7 vs 11.2, *p* = 0.459; HADS-depression: 11.6 vs 10.7, *p* = 0.656). Patients with ILD-GAP ≥4 had lower FVC and DLCO, and those with CFS ≥5 additionally had more dyspnoea and greater oxygen and opioid use; neither group differed in baseline HADS (Supplementary Tables 3 and 4) or in response (Figure 3).

### Patient- and caregiver-reported experience

PREMs were high across all domains (Table 2), with mean scores ranging from 9.1 to 10.0 in patients and from 8.8 to 9.9 in caregivers. Patient and caregiver ratings were strongly correlated (total scores, *n* = 18; Spearman’s rho = 0.836, *p* < 0.001).

**Table 2.** Patient- and caregiver-reported experience measures (PREMs).

Mean (SD)	Patients (*N* = 24)	Caregivers (*N* = 18)
Perception that the intervention helped address the initial concern or distress	9.5 (0.7)	9.6 (0.8)
Satisfaction with the professional’s support throughout the process	9.1 (1.1)	8.8 (1.5)
Perception that the professional provided useful tools to better cope with the disease	9.5 (0.7)	9.7 (0.6)
Perceived improvement in emotional well-being after psychological care	9.3 (1.1)	9.2 (1.2)
Perception that the professional cared about the patient’s well-being and treated them with respect	9.1 (1.2)	8.9 (1.6)
Belief that psychological care improves the well-being of people living with a chronic disease	9.8 (0.5)	9.9 (0.2)
Likelihood of recommending the service to others	9.4 (0.8)	9.7 (0.6)
Overall satisfaction with the psychological care provided	10.0 (0.2)	9.9 (0.5)
**Total score**	**9.4 (0.7)**	**9.5 (0.7)**

Values are mean (standard deviation) on a 0–10 scale.

PREMs: patient- and caregiver-reported experience measures. SD: standard deviation.

## Discussion

In this single-arm before-and-after study, an individualised psychological intervention was associated with clinically meaningful reductions in HADS-anxiety and HADS-depression scores, and no patient worsened in HADS category. A higher baseline HADS score was the only predefined characteristic associated with improvement in HADS category; no differences in response were detected across disease severity, frailty, time since diagnosis, age or sex. However, the uncontrolled design and the exploratory nature of these analyses limit interpretation. Within these limits, the absence of such differences suggests that eligibility for psychological care should not be restricted by disease stage, functional impairment or time since diagnosis.

The high proportion of patients scoring ≥8 on each HADS subscale at baseline (76.7%) likely reflects the enriched referral approach rather than population prevalence [14–19]. A documented history of pre-existing anxiety and/or depression was present in 23.3% of patients and did not predict higher baseline HADS scores, consistent with the observation that emotional distress in fibrotic ILD may develop with the disease rather than reflect a pre-existing vulnerability, with dyspnoea and cough reported as major contributors [19,34]. The observed reductions of 4.7 and 5.2 points exceeded both the 1.7-point threshold applied in this study [32] and the IPF-specific estimates of −2 for anxiety and −1.2 for depression [33] by more than twofold, and were substantially greater than the random within-subject variability expected for the instrument. In addition, baseline HADS scores in our cohort were comparable to those previously reported in patients with IPF and mood disorders (9.1–11.2), and the HADS has high test–retest reliability (0.86). Together, these findings make it unlikely that regression to the mean or natural fluctuation alone explains the observed group-level effect [33]. Within the cohort, however, patients with higher baseline scores had more room to improve, and those in the normal range at baseline could not, by definition, move to a less severe category; this association is therefore partly attributable to regression to the mean or a floor effect and does not establish greater responsiveness; whether they derive greater clinical benefit requires controlled studies.

The reductions in HADS-anxiety and HADS-depression scores are consistent with previous interventional evidence in ILD, although direct comparisons are limited by differences in design, intervention modality, and outcome measures. The TIRED trial [20] reported that a 12-week online mindfulness-based cognitive therapy programme reduced fatigue, the primary endpoint, and also improved anxiety and depression as pre-specified secondary outcomes in patients with sarcoidosis, and the COMPANION trial [18] showed that a 9-week digital cognitive behavioural therapy (CBT) reduced anxiety measured with the Generalised Anxiety Disorder-7 (GAD-7) scale in patients with pulmonary fibrosis. The ADAPT trial [35] evaluated a nurse- and social-worker-led palliative telecare programme across COPD, heart failure, and ILD, in which ILD patients comprised only 4% of the cohort; the programme improved quality of life and reduced depression and anxiety. In advanced cancer, the CALM trial [36] showed that a clinician-delivered, semi-structured psychological intervention addressing existential concerns reduced depressive symptoms and death-related distress. The absence of worsening in HADS category in our cohort is notable when set against the natural history of emotional distress in pulmonary fibrosis. In the Australian IPF Registry [34], where patients were followed under usual care, 13% of patients showed worsening anxiety and 7% worsening depression at 12-month follow-up. The contrast with our cohort could be consistent with a stabilising effect of the intervention, although the absence of a control arm precludes causal attribution.

The largest trial of psychological intervention in chronic respiratory disease, TANDEM [23], found no benefit of a fixed 6–8-week CBT intervention delivered by respiratory healthcare professionals trained in brief CBT in COPD. By contrast, a meta-analysis of 35 randomised controlled trials in COPD reported overall benefit of psychosocial interventions on physical and psychological outcomes [37]. In our study, the intervention was delivered by a psychologist experienced in psycho-oncology and palliative care using an integrative model incorporating meaning-centred psychotherapy [29], which has been shown to improve a sense of meaning and spiritual wellbeing and to reduce anxiety and the desire for hastened death in advanced cancer, alongside acceptance and commitment therapy, which reduced anxiety and depression in a meta-analysis of 16 randomised trials in cancer [38]. Sessions were not scheduled at fixed intervals but according to psychological need. The integrative model may underlie the consistency of response across patient profiles. The progressive trajectory, prognostic uncertainty and existential burden of pulmonary fibrosis parallel those of advanced malignancy [2], and may not be adequately addressed by standard CBT, which may support the transposition of psycho-oncology frameworks to fibrotic ILD.

The high frequency of coping styles classified as maladaptive in our cohort (93.3%) is consistent with selection by clinical distress and parallels patterns described in advanced cancer, where avoidance-oriented coping has been associated with worse psychological outcomes [39]. In oncology, systematic distress screening and psychological referral are embedded across the disease continuum [40]. Our programme delivered an integrated, collaborative model within routine ILD care, with case discussion with the treating pulmonologist and ILD nurse specialist as needed; such models have shown benefit on both depressive symptoms and overall illness burden in patients with chronic medical conditions [41]. Referral in our programme was triggered by clinically suspected distress, that is, targeted intervention rather than universal support. Whether universal screening would identify additional patients who benefit remains open, since routine distress screening in oncology is feasible when linked to specialist pathways but its overall effectiveness is not established [42].

PREMs were high for both patients and caregivers, with mean scores ≥8.8 across all domains. These measures reflect acceptability and perceived value rather than effectiveness, and the two items on perceived emotional improvement are perceptions, not outcomes. With these caveats, psychological care delivered in an ILD clinic was well accepted by patients and caregivers, and no patient discontinued for reasons other than intercurrent clinical events. This is relevant because psychological support is often assumed to be of limited interest to these patients, and reluctance and stigma towards psychological care may limit uptake [43]. The strong patient–caregiver correlation reflects consistency between informants rather than validity of an instrument developed ad hoc and not formally validated.

### Limitations and strengths

Several limitations warrant consideration. The single-arm before-and-after design and limited sample size constrain causal inference, and natural mood fluctuation, regression to the mean or non-specific effects of therapeutic attention cannot be excluded. The subgroup analyses were defined before any analysis of outcome data, and the study was not powered for them. The number of patients offered or declining referral is unknown and the proportion of the ILD population represented cannot be quantified; enriched referral of patients with suspected distress inflated baseline severity relative to unselected ILD populations, but reflects the clinical scenario in which referral to psychology is actually triggered, so our findings are generalisable only to patients in whom emotional distress is clinically suspected; moreover, the intervention was delivered in a specialist ILD service by a single psychologist experienced in psycho-oncology and palliative care, and the findings may not extend to unselected fibrotic ILD populations, to services with different referral pathways or to other therapists. The loss of six patients (20.0%) is an important limitation. Discontinuation was not due to withdrawal or loss of adherence but to intercurrent clinical events, and in these patients, whose disease had progressed, a favourable psychological outcome cannot be assumed. These data are therefore unlikely to be missing at random, and the sensitivity analysis accordingly imputed deterioration in these patients, with statistical significance lost beyond a post-intervention HADS score of 18. HADS was self-reported and was not complemented by structured psychiatric evaluation or diagnostic interview, so the findings describe symptom burden and risk rather than diagnosed disorders; a potential social-desirability bias cannot be excluded. The intervention was individualised and not manualised, and the number, timing and content of sessions were determined clinically, which limits standardisation and precludes formal dose-response analysis. Coping was clinically classified rather than psychometrically scored, precluding inter-rater reliability assessment; classification by the clinician delivering the intervention may additionally have introduced rating bias. The PREM questionnaire was developed *ad hoc* for this study and lacks formal psychometric validation. Finally, outcomes were captured at discharge or closure, and durability beyond treatment remains unknown.

Notable strengths include the real-world implementation of a structured but individualised intervention by a psychologist experienced in psycho-oncology and palliative care, with disease-specific content applied across the full spectrum of disease severity, the consistency of delivery ensured by a single-centre design, and high patient- and caregiver-reported experience scores.

### Future directions

Multicentre randomised trials should define optimal intervention timing, whether at diagnosis, at clinical milestones or when distress emerges. They should also compare delivery models, including a direct comparison of universal distress screening with targeted referral. Integration of psycho-oncology pathways into ILD services and psychometric validation of coping inventories warrant formal evaluation.

## Conclusion

In this study, an individualised, integrative psychological intervention was associated with reduced anxiety and depressive symptoms and a lower proportion of patients at risk of clinically relevant anxiety and depression in pulmonary fibrosis, with high patient- and caregiver-reported experience scores and no worsening in HADS category. Given the uncontrolled, single-centre and exploratory nature of the study, these findings are hypothesis-generating, and any implications for clinical practice should be regarded as preliminary and require confirmation in larger multicentre randomised trials.

## Supplementary Material

Supplementary_Material_reviewed_clean.docx

## Data Availability

The datasets analysed during the current study are available from the corresponding author on reasonable request.
