# Supplementary material for: The potential of psychological intervention to decrease the risk of anxiety and depression in pulmonary fibrosis
Source: Ann Med. 2026 Oct 22;58(1):2743870. doi: 10.1080/07853890.2026.2743870 (PMC13700519; doi:10.1080/07853890.2026.2743870)
Supplement: Supplementary file 1 [file IANN_A_2743870_SM8698.docx]

**Supplementary Material**

**Sample size**

The primary analysis compares pre- and post-intervention HADS-anxiety and HADS-depression scores. A formal sample size calculation was performed assuming a two-sided paired t-test. To control the family-wise type I error rate across the two primary endpoints, the nominal significance level was set at α=0.05 and split equally (α=0.025 per endpoint).

A minimal important difference of 1.7 points (Smid, 2017, PMID 27624705) was assumed, with a standard deviation of 3.4 and a within-subject correlation of 0.72 (Nikolovski, 2024, PMID 36861817). Under these assumptions, 24 patients were required to achieve 80% power (Cohen, 1988).

Allowing for an anticipated 20% dropout rate (Smid, 2017, PMID 27624705; Oriani, 2020, PMID 31918702), the final target sample size was 30 patients. Sample size calculations were performed in R (version 4.5) using the *pwr* package and function pwr.t.test().

**R code for sample size estimation**

**1. Package Installation and Setup**

install.packages("pwr");

library(pwr)

**2. Assumptions**

delta <- 1.7	# Minimal important difference

sd <- 3.4	# Pooled standard deviation

r <- 0.72	# Within-subject Pearson correlation

drop <- 0.20	# Expected drop-out rate

**3. Standardized effect size for paired samples**

d <- delta / (sd * sqrt(2 * (1 - r)))

**4. Required sample size for paired t-test**

ss <- pwr.t.test( d = d, power = 0.80, sig.level = 0.025, type = "paired", alternative = "two.sided" )

**5. Inflation for anticipated drop-out**

n_total <- ceiling(ss$n / (1 - drop))

n_total

**6. Software**

All analyses were performed using R (version 4.5).

**Statistical analysis**

The primary analysis compared pre- and post-intervention HADS-anxiety and HADS-depression scores using paired data from patients with complete assessments. The total two-sided type I error rate of 0.05 was allocated equally between the two primary outcomes, yielding a significance threshold of 0.025 per outcome. A sample size of 30 patients was planned (see Sample Size section above).

Pre- and post-intervention changes in HADS scores were analysed using paired t-test mean differences and associated 97.5% confidence intervals. The assumption of normality of the paired differences was assessed by means of the Shapiro–Wilk test. As a complementary non-parametric approach, the Wilcoxon signed-rank test was applied, with median differences and their 97.5% confidence intervals estimated using the Hodges–Lehmann location-shift estimator.

A sensitivity analysis was conducted to assess the robustness of the results to potentially informative missingness due to intercurrent events (death, palliative home care, or transplantation), which are likely associated with clinical deterioration. Post-intervention HADS scores for patients with missing follow-up data were imputed with values ranging from their observed baseline score to the maximum possible score (21 points), and the t-test was re-applied at each imputed value. The maximum deterioration from baseline immediately prior to the loss of statistical significance (p ≥ 0.025) was reported as the tipping point, following the tipping-point approach to sensitivity analysis under informative missingness (Liublinska, 2014, PMID 24845086; Torres, 2025, PMID 40140629). Tipping-point analyses have also been applied to evidence that includes single-arm studies (Han, 2024, PMID 39054412).

Subgroup analyses were conducted to describe treatment response across patient subgroups. For the subgroup and regression analyses, patients were classified as improved if they shifted to a less severe HADS category between baseline and post-intervention assessment; the descriptive count reported in the main text instead classifies patients by the direction of change in the subscale score. Subgroups were defined before any analysis of outcome data was undertaken, on the basis of clinical judgement, as those most likely to influence response to the psychological intervention: age (≥75 years), sex, disease severity (ILD-GAP <4 vs ≥4; CFS <5 vs ≥5), time since diagnosis (>12 months), and baseline HADS levels. Logistic regression models were used to estimate odds ratios and corresponding 95% confidence intervals based on asymptotic standard errors. Statistical significance was assessed using two-sided Wald tests.

Patient and caregiver PREMs were compared using Spearman's rank correlation coefficient.

Associations between patient characteristics and changes in HADS scores from baseline to post-intervention (improved vs. not improved), as well as disease severity (ILD-GAP <4 vs ≥4; CFS <5 vs ≥5), were assessed using chi-square or Fisher’s exact tests for categorical variables, and the Student’s t-test or Mann–Whitney U test for continuous variables, as appropriate. Non-parametric and exact methods were used when assumptions of parametric tests were not met.

Multivariable logistic regression was used to estimate independent associations between baseline characteristics and improvement. Model specification was guided by clinical relevance and Akaike’s information criterion (AIC). Model discrimination was assessed using the area under the receiver operating characteristic curve (AUC), and internal validation was performed using repeated 5-fold cross-validation (10 repetitions).

For secondary and exploratory analyses, we used two-sided p values with an α level of significance of 0.05 or less, provided that both primary outcomes reached statistical significance at the 0.025 threshold. No adjustment for multiple comparisons was applied, as secondary analyses were considered supportive and exploratory analyses were considered hypothesis-generating rather than confirmatory. Accordingly, p-values from these analyses should be interpreted as descriptive. Statistical analyses were performed using R (version 4.5).

**R code for primary analyses of HADS scores**

**1. Package Installation and Setup**

install.packages("DescTools")

library(DescTools)

**2. Data Preparation**

The dataset HAD contains one row per patient:

\# HAD$anspre = Anxiety score pre-intervention

\# HAD$anspost = Anxiety score post-intervention

\# HAD$deppre = Depression score pre-intervention

\# HAD$deppost = Depression score post-intervention

**3. Primary Analysis: Paired Student’s t-test**

A two-sided paired t-test was used as the primary analysis. The significance level was set at α = 0.025 (two-sided); accordingly, 97.5% confidence intervals are reported (see Sample Size section above).

######### Anxiety subscale #########################################

\## Paired t-test

t.test(x	= HAD$anspre,

y	= HAD$anspost,

alternative = "two.sided",

paired	= TRUE,

conf.level = 0.975)

######### Depression subscale ######################################

\## Paired t-test

t.test(x	= HAD$deppre,

y	= HAD$deppost,

alternative = "two.sided",

paired	= TRUE,

conf.level = 0.975)

**4. Normality Assessment**

The Shapiro–Wilk test was applied to the paired differences to assess the normality assumption of the paired Student's t-test.

######### Anxiety subscale #########################################

\## Shapiro-Wilk normality test on paired differences

shapiro.test(HAD$anspre - HAD$anspost)

######### Depression subscale ######################################

\## Shapiro-Wilk normality test on paired differences

shapiro.test(HAD$deppre - HAD$deppost)

**5. Complementary Non-parametric Analysis**

The Wilcoxon signed-rank test was applied as a complementary non-parametric analysis. Median differences and their 97.5% confidence intervals were estimated using the Hodges–Lehmann location-shift estimator. Exact p-values were not computed due to the presence of ties in the paired differences; a normal approximation was used instead.

######### Anxiety subscale #########################################

\## Wilcoxon signed-rank test with Hodges-Lehmann location-shift estimator

wilcox.test(x	= HAD$anspre,

y	= HAD$anspost,

alternative	= "two.sided",

paired	= TRUE,

conf.int	= TRUE,

conf.level	= 0.975,

exact	= FALSE)

######### Depression subscale ######################################

\## Wilcoxon signed-rank test with Hodges-Lehmann location-shift estimator

wilcox.test(x	= HAD$deppre,

y	= HAD$deppost,

alternative = "two.sided",

paired	= TRUE,

conf.int	= TRUE,

conf.level	= 0.975,

exact	= FALSE)

**6. Software**

All analyses were performed using R (version 4.5).

**Supplementary Figure 1.** HADS-anxiety and HADS-depression subscale scores before and after the psychological intervention — sensitivity analysis (n=30). HADS score thresholds: 0–7 normal, 8–10 mild, 11–14 moderate, 15–21 severe. **HADS**: Hospital Anxiety and Depression Scale. In the sensitivity analysis, the six patients who discontinued before post-intervention evaluation were assigned a post-intervention HADS score of 18, the tipping point value immediately prior to losing statistical significance (p ≥ 0.025).

Pre- and post-intervention changes in HADS scores were analysed using paired t-test mean differences and associated 97.5% confidence intervals. The p-values from the Shapiro–Wilk test for the differences in HADS-anxiety and HADS-depression scores were 0.526 and 0.276, respectively.

**Supplementary Table 1.** Baseline characteristics compared between patients with and without improvement in HADS-anxiety category. **ILD**: interstitial lung disease. **HP**: hypersensitivity pneumonitis. **CTD-ILD**: connective tissue disease-associated ILD. **IPAF**: interstitial pneumonia with autoimmune features. **FVC**: forced vital capacity (% predicted). **DLCO**: diffusing capacity for carbon monoxide (% predicted). **ILD-GAP**: ILD-Gender, Age, Physiology index. **CFS**: Clinical Frailty Scale. **mMRC**: modified Medical Research Council dyspnoea scale. **HADS**: Hospital Anxiety and Depression Scale. **SD**: standard deviation. Baseline HADS-anxiety score was the only characteristic retained in the multivariable model; cross-validated AUC 94.7% (95% CI: 93.3–96.0%). AUC: area under the receiver operating characteristic curve.

Footnotes: ᵃ Documented in primary care records without formal psychiatric or psychological evaluation; ^b^ Clinically assigned by the treating psychologist; not psychometrically scored (see Methods).

| Characteristics | Evaluable patients,  N=24 | Improved,  n=14 | Not improved,  n=10 | p-value |
| --- | --- | --- | --- | --- |
| **Sociodemographic characteristics** |  |  |  |  |
| Male, n (%) | 11 (45.8%) | 7 (50.0%) | 4 (40.0%) | 0.697 |
| Age, mean (SD) | 71.2 (8.5) | 73.3 (6.6) | 68.3 (10.3) | 0.212 |
| Civil status, n (%) |  |  |  | 0.203 |
| Married | 17 (70.8%) | 10 (71.4%) | 7 (70.0%) |  |
| Widowed | 5 (20.8%) | 4 (28.6%) | 1 (10.0%) |  |
| Divorced | 1 (4.2%) | 0 (0.0%) | 1 (10.0%) |  |
| Single | 1 (4.2%) | 0 (0.0%) | 1 (10.0%) |  |
| Support network available, n (%) | 23 (95.8%) | 14 (100.0%) | 9 (90.0%) | 0.417 |
| Effective support network, n (%) | 19 (79.2%) | 12 (85.7%) | 7 (70.0%) | 0.615 |
| **Disease and clinical characteristics** |  |  |  |  |
| ILD diagnosis, n (%) |  |  |  | 0.473 |
| Chronic HP | 9 (37.5%) | 6 (42.9%) | 3 (30.0%) |  |
| IPF | 6 (25.0%) | 4 (28.6%) | 2 (20.0%) |  |
| CTD-ILD | 4 (16.7%) | 3 (21.4%) | 1 (10.0%) |  |
| IPAF | 3 (12.5%) | 1 (7.1%) | 2 (20.0%) |  |
| Other | 2 (8.3%) | 0 (0.0%) | 2 (20.0%) |  |
| FVC (% of predicted), mean (SD) | 75.2 (20.9) | 74.7 (18.8) | 76.1 (24.6) | 0.796 |
| DLCO (% of predicted), mean (SD) | 53.2 (13.4) | 50.6 (15.0) | 57.0 (10.7) | 0.315 |
| ILD-GAP index ≥4, n (%) | 12 (50.0%) | 7 (50.0%) | 5 (50.0%) | >0.999 |
| CFS ≥5, n (%) | 8 (33.3%) | 6 (42.9%) | 2 (20.0%) | 0.388 |
| Time since diagnosis in months, mean (SD) | 18.0 (20.0) | 15.5 (11.7) | 21.6 (28.3) | 0.796 |
| ≤6 months, n (%) | 6 (25.0%) | 4 (28.6%) | 2 (20.0%) | >0.999 |
| ≤12 months, n (%) | 12 (50.0%) | 6 (42.9%) | 6 (60.0%) | 0.408 |
| Dyspnoea (mMRC), n (%) |  |  |  | 0.497 |
| Grade 1 | 11 (45.8%) | 8 (57.1%) | 3 (30.0%) |  |
| Grade 2 | 7 (29.2%) | 3 (21.4%) | 4 (40.0%) |  |
| Grade 3 | 6 (25.0%) | 3 (21.4%) | 3 (30.0%) |  |
| Cough, n (%) | 10 (41.7%) | 6 (42.9%) | 4 (40.0%) | >0.999 |
| Family aggregation, n (%) | 5 (20.8%) | 4 (28.6%) | 1 (10.0%) | 0.358 |
| **Treatment and supportive care** |  |  |  |  |
| Antifibrotic therapy, n (%) | 23 (95.8%) | 13 (92.9%) | 10 (100.0%) | >0.999 |
| Antifibrotic side effects, n (%) | 4 (16.7%) | 1 (7.1%) | 3 (30.0%) | 0.272 |
| O2 on exertion, n (%) | 8 (33.3%) | 4 (28.6%) | 4 (40.0%) | 0.673 |
| O2 at rest, n (%) | 3 (12.5%) | 2 (14.3%) | 1 (10.0%) | >0.999 |
| Opioid use, n (%) | 6 (25.0%) | 5 (35.7%) | 1 (10.0%) | 0.341 |
| Lung transplant evaluation, n (%) | 4 (16.7%) | 2 (14.3%) | 2 (20.0%) | >0.999 |
| **Psychological history and baseline status** |  |  |  |  |
| Anxiety and/or depression (primary care record)^a^, n (%) | 6 (25.0%) | 5 (35.7%) | 1 (10.0%) | 0.341 |
| Fibromyalgia, n (%) | 3 (12.5%) | 3 (21.4%) | 0 (0.0%) | 0.239 |
| Bereavement, n (%) | 7 (29.2%) | 5 (35.7%) | 2 (20.0%) | 0.653 |
| Predominant concern, n (%) |  |  |  | 0.855 |
| Loss of autonomy | 8 (33.3%) | 5 (35.7%) | 3 (30.0%) |  |
| Disease progression or death | 7 (29.2%) | 5 (35.7%) | 2 (20.0%) |  |
| Secondary losses | 2 (8.3%) | 1 (7.1%) | 1 (10.0%) |  |
| Treatment tolerance | 2 (8.3%) | 1 (7.1%) | 1 (10.0%) |  |
| Other | 5 (20.8%) | 2 (14.3%) | 3 (30.0%) |  |
| Information integration, n (%) |  |  |  | 0.358 |
| Full | 19 (79.2%) | 10 (71.4%) | 9 (90.0%) |  |
| Partial | 5 (20.8%) | 4 (28.6%) | 1 (10.0%) |  |
| Coping style at baseline^b^, n (%) |  |  |  | 0.206 |
| Anxious preoccupation | 7 (29.2%) | 6 (42.9%) | 1 (10.0%) |  |
| Fatalism | 8 (33.3%) | 5 (35.7%) | 3 (30.0%) |  |
| Cognitive avoidance | 5 (20.8%) | 2 (14.3%) | 3 (30.0%) |  |
| Helplessness-hopelessness | 2 (8.3%) | 1 (7.1%) | 1 (10.0%) |  |
| Fighting spirit | 2 (8.3%) | 0 (0.0%) | 2 (20.0%) |  |
| **HADS baseline** |  |  |  |  |
| Anxiety subscale, mean (SD) | 11.3 (4.8) | 14.4 (3.4) | 7.1 (2.9) | <0.001 |
| Anxiety level, n (%) |  |  |  | <0.001 |
| Severe | 8 (33.3%) | 8 (57.1%) | 0 (0.0%) |  |
| Moderate | 6 (25.0%) | 4 (28.6%) | 2 (20.0%) |  |
| Mild | 3 (12.5%) | 2 (14.3%) | 1 (10.0%) |  |
| Normal | 7 (29.2%) | 0 (0.0%) | 7 (70.0%) |  |
| Depression subscale, mean (SD) | 10.7 (4.6) | 13.2 (3.8) | 7.2 (3.0) | <0.001 |
| Depression level, n (%) |  |  |  | 0.002 |
| Severe | 4 (16.7%) | 4 (28.6%) | 0 (0.0%) |  |
| Moderate | 9 (37.5%) | 8 (57.1%) | 1 (10.0%) |  |
| Mild | 5 (20.8%) | 1 (7.1%) | 4 (40.0%) |  |
| Normal | 6 (25.0%) | 1 (7.1%) | 5 (50.0%) |  |
| Data are presented as percentages by column. | | | | |

**Supplementary Table 2.** Baseline characteristics compared between patients with and without improvement in HADS-depression category. **ILD**: interstitial lung disease. **HP**: hypersensitivity pneumonitis. **CTD-ILD**: connective tissue disease-associated ILD. **IPAF**: interstitial pneumonia with autoimmune features. **FVC**: forced vital capacity (% predicted). **DLCO**: diffusing capacity for carbon monoxide (% predicted). **ILD-GAP**: ILD-Gender, Age, Physiology index. **CFS**: Clinical Frailty Scale. **mMRC**: modified Medical Research Council dyspnoea scale. **HADS: Hospital Anxiety and Depression Scale. SD: standard deviation. Baseline HADS-depression score was the only characteristic retained in the multivariable model; cross-validated AUC 83.8% (95% CI: 80.8–86.7%). AUC: area under the receiver operating characteristic curve.**

Footnotes: ᵃ Documented in primary care records without formal psychiatric or psychological evaluation; ^b^ Clinically assigned by the treating psychologist; not psychometrically scored (see Methods).

| Characteristics | Evaluable patients,  N=24 | Improved,  n=15 | Not improved,  n=9 | p-value |
| --- | --- | --- | --- | --- |
| **Sociodemographic characteristics** |  |  |  |  |
| Male, n (%) | 11 (45.8%) | 6 (40.0%) | 5 (55.6%) | 0.675 |
| Age, mean (SD) | 71.2 (8.5) | 71.0 (9.6) | 71.5 (7.0) | 0.907 |
| Civil status, n (%) |  |  |  | 0.113 |
| Married | 17 (70.8%) | 9 (60.0%) | 8 (88.9%) |  |
| Widowed | 5 (20.8%) | 5 (33.3%) | 0 (0.0%) |  |
| Divorced | 1 (4.2%) | 0 (0.0%) | 1 (11.1%) |  |
| Single | 1 (4.2%) | 1 (6.7%) | 0 (0.0%) |  |
| Support network available, n (%) | 23 (95.8%) | 15 (100.0%) | 8 (88.9%) | 0.375 |
| Effective support network, n (%) | 19 (79.2%) | 12 (80.0%) | 7 (77.8%) | >0.999 |
| **Disease and clinical characteristics** |  |  |  |  |
| ILD diagnosis, n (%) |  |  |  | 0.330 |
| Chronic HP | 9 (37.5%) | 5 (33.3%) | 4 (44.4%) |  |
| IPF | 6 (25.0%) | 3 (20.0%) | 3 (33.3%) |  |
| CTD-ILD | 4 (16.7%) | 4 (26.7%) | 0 (0.0%) |  |
| IPAF | 3 (12.5%) | 1 (6.7%) | 2 (22.2%) |  |
| Other | 2 (8.3%) | 2 (13.3%) | 0 (0.0%) |  |
| FVC (% of predicted), mean (SD) | 75.2 (20.9) | 72.4 (20.1) | 79.9 (22.6) | 0.411 |
| DLCO (% of predicted), mean (SD) | 53.2 (13.4) | 51.8 (14.8) | 55.3 (11.9) | 0.601 |
| ILD-GAP index ≥4, n (%) | 12 (50.0%) | 7 (46.7%) | 5 (55.6%) | >0.999 |
| CFS ≥5, n (%) | 8 (33.3%) | 6 (40.0%) | 2 (22.2%) | 0.657 |
| Time since diagnosis in months, mean (SD) | 18.0 (20.0) | 20.2 (21.7) | 14.4 (17.4) | 0.290 |
| ≤6 months, n (%) | 6 (25.0%) | 3 (20.0%) | 3 (33.3%) | 0.635 |
| ≤12 months, n (%) | 12 (50.0%) | 7 (46.7%) | 5 (55.6%) | >0.999 |
| Dyspnoea (mMRC), n (%) |  |  |  | 0.488 |
| Grade 1 | 11 (45.8%) | 8 (53.3%) | 3 (33.3%) |  |
| Grade 2 | 7 (29.2%) | 3 (20.0%) | 4 (44.4%) |  |
| Grade 3 | 6 (25.0%) | 4 (26.7%) | 2 (22.2%) |  |
| Cough, n (%) | 10 (41.7%) | 6 (40.0%) | 4 (44.4%) | >0.999 |
| Family aggregation, n (%) | 5 (20.8%) | 5 (33.3%) | 0 (0.0%) | 0.118 |
| **Treatment and supportive care** |  |  |  |  |
| Antifibrotic therapy, n (%) | 23 (95.8%) | 14 (93.3%) | 9 (100.0%) | >0.999 |
| Antifibrotic side effects, n (%) | 4 (16.7%) | 1 (6.7%) | 3 (33.3%) | 0.130 |
| O2 on exertion, n (%) | 8 (33.3%) | 4 (26.7%) | 4 (44.4%) | 0.412 |
| O2 at rest, n (%) | 3 (12.5%) | 2 (13.3%) | 1 (11.1%) | >0.999 |
| Opioid use, n (%) | 6 (25.0%) | 5 (33.3%) | 1 (11.1%) | 0.351 |
| Lung transplant evaluation, n (%) | 4 (16.7%) | 3 (20.0%) | 1 (11.1%) | >0.999 |
| **Psychological history and baseline status** |  |  |  |  |
| Anxiety and/or depression (primary care record)^a^, n (%) | 6 (25.0%) | 5 (33.3%) | 1 (11.1%) | 0.351 |
| Fibromyalgia, n (%) | 3 (12.5%) | 2 (13.3%) | 1 (11.1%) | >0.999 |
| Bereavement, n (%) | 7 (29.2%) | 7 (46.7%) | 0 (0.0%) | 0.022 |
| Predominant concern, n (%) |  |  |  | 0.964 |
| Loss of autonomy | 8 (33.3%) | 6 (40.0%) | 2 (22.2%) |  |
| Disease progression or death | 7 (29.2%) | 4 (26.7%) | 3 (33.3%) |  |
| Secondary losses | 2 (8.3%) | 1 (6.7%) | 1 (11.1%) |  |
| Treatment tolerance | 2 (8.3%) | 1 (6.7%) | 1 (11.1%) |  |
| Other | 5 (20.8%) | 3 (20.0%) | 2 (22.2%) |  |
| Information integration, n (%) |  |  |  | 0.118 |
| Full | 19 (79.2%) | 10 (66.7%) | 9 (100.0%) |  |
| Partial | 5 (20.8%) | 5 (33.3%) | 0 (0.0%) |  |
| Coping style at baseline^b^, n (%) |  |  |  | 0.844 |
| Anxious preoccupation | 7 (29.2%) | 5 (33.3%) | 2 (22.2%) |  |
| Fatalism | 8 (33.3%) | 4 (26.7%) | 4 (44.4%) |  |
| Cognitive avoidance | 5 (20.8%) | 4 (26.7%) | 1 (11.1%) |  |
| Helplessness-hopelessness | 2 (8.3%) | 1 (6.7%) | 1 (11.1%) |  |
| Fighting spirit | 2 (8.3%) | 1 (6.7%) | 1 (11.1%) |  |
| **HADS baseline** |  |  |  |  |
| Anxiety subscale, mean (SD) | 11.3 (4.8) | 13.4 (4.3) | 7.9 (3.5) | 0.007 |
| Anxiety level, n (%) |  |  |  | 0.019 |
| Severe | 8 (33.3%) | 8 (53.3%) | 0 (0.0%) |  |
| Moderate | 6 (25.0%) | 3 (20.0%) | 3 (33.3%) |  |
| Mild | 3 (12.5%) | 2 (13.3%) | 1 (11.1%) |  |
| Normal | 7 (29.2%) | 2 (13.3%) | 5 (55.6%) |  |
| Depression subscale, mean (SD) | 10.7 (4.6) | 12.6 (3.2) | 7.6 (4.9) | 0.008 |
| Depression level, n (%) |  |  |  | 0.002 |
| Severe | 4 (16.7%) | 3 (20.0%) | 1 (11.1%) |  |
| Moderate | 9 (37.5%) | 8 (53.3%) | 1 (11.1%) |  |
| Mild | 5 (20.8%) | 4 (26.7%) | 1 (11.1%) |  |
| Normal | 6 (25.0%) | 0 (0.0%) | 6 (66.7%) |  |
| Data are presented as percentages by column. | | | | |

**Supplementary Table 3.** Baseline characteristics compared between patients with ILD-GAP <4 and ILD-GAP ≥4. **ILD**: interstitial lung disease. **HP**: hypersensitivity pneumonitis. **CTD-ILD**: connective tissue disease-associated ILD. **IPAF**: interstitial pneumonia with autoimmune features. **FVC**: forced vital capacity (% predicted). **DLCO**: diffusing capacity for carbon monoxide (% predicted). **ILD-GAP**: ILD-Gender, Age, Physiology index. **CFS**: Clinical Frailty Scale. **mMRC**: modified Medical Research Council dyspnoea scale. **HADS**: Hospital Anxiety and Depression Scale. **SD**: standard deviation.

Footnotes: ᵃ Documented in primary care records without formal psychiatric or psychological evaluation; ^b^ Clinically assigned by the treating psychologist; not psychometrically scored (see Methods).

| Characteristics | All patients,  N=30 | ILD-GAP <4,  n=13 | ILD-GAP ≥4,  n=17 | p-value |
| --- | --- | --- | --- | --- |
| **Sociodemographic characteristics** |  |  |  |  |
| Male, n (%) | 15 (50.0%) | 6 (46.2%) | 9 (52.9%) | 0.713 |
| Age, mean (SD) | 71.0 (8.3) | 71.4 (8.5) | 70.7 (8.4) | 0.967 |
| Civil status, n (%) |  |  |  | 0.147 |
| Married | 22 (73.3%) | 12 (92.3%) | 10 (58.8%) |  |
| Widowed | 6 (20.0%) | 1 (7.7%) | 5 (29.4%) |  |
| Divorced | 1 (3.3%) | 0 (0.0%) | 1 (5.9%) |  |
| Single | 1 (3.3%) | 0 (0.0%) | 1 (5.9%) |  |
| Support network available, n (%) | 29 (96.7%) | 13 (100.0%) | 16 (94.1%) | >0.999 |
| Effective support network, n (%) | 24 (80.0%) | 12 (92.3%) | 12 (70.6%) | 0.196 |
| **Disease and clinical characteristics** |  |  |  |  |
| ILD diagnosis, n (%) |  |  |  | 0.845 |
| Chronic HP | 11 (36.7%) | 6 (46.2%) | 5 (29.4%) |  |
| IPF | 10 (33.3%) | 3 (23.1%) | 7 (41.2%) |  |
| CTD-ILD | 4 (13.3%) | 2 (15.4%) | 2 (11.8%) |  |
| IPAF | 3 (10.0%) | 1 (7.7%) | 2 (11.8%) |  |
| Other | 2 (6.7%) | 1 (7.7%) | 1 (5.9%) |  |
| FVC (% of predicted), mean (SD) | 72.4 (20.9) | 85.8 (11.9) | 62.2 (20.7) | 0.001 |
| DLCO (% of predicted), mean (SD) | 48.7 (15.4) | 59.0 (12.5) | 37.5 (9.4) | <0.001 |
| CFS ≥5, n (%) | 14 (46.7%) | 2 (15.4%) | 12 (70.6%) | 0.003 |
| Time since diagnosis in months, mean (SD) | 18.7 (19.2) | 13.5 (12.1) | 22.7 (22.7) | 0.245 |
| ≤6 months, n (%) | 8 (26.7%) | 4 (30.8%) | 4 (23.5%) | 0.698 |
| ≤12 months, n (%) | 14 (46.7%) | 7 (53.8%) | 7 (41.2%) | 0.491 |
| Dyspnoea (mMRC), n (%) |  |  |  | 0.062 |
| Grade 1 | 11 (36.7%) | 7 (53.8%) | 4 (23.5%) |  |
| Grade 2 | 7 (23.3%) | 4 (30.8%) | 3 (17.6%) |  |
| Grade 3 | 12 (40.0%) | 2 (15.4%) | 10 (58.8%) |  |
| Family aggregation, n (%) | 6 (20.0%) | 6 (46.2%) | 0 (0.0%) | 0.003 |
| **Treatment and supportive care** |  |  |  |  |
| Antifibrotic therapy, n (%) | 27 (90.0%) | 12 (92.3%) | 15 (88.2%) | >0.999 |
| Antifibrotic side effects, n (%) | 4 (13.3%) | 4 (30.8%) | 0 (0.0%) | 0.026 |
| O2 on exertion, n (%) | 13 (43.3%) | 4 (30.8%) | 9 (52.9%) | 0.225 |
| O2 at rest, n (%) | 4 (13.3%) | 2 (15.4%) | 2 (11.8%) | >0.999 |
| Opioid use, n (%) | 11 (36.7%) | 4 (30.8%) | 7 (41.2%) | 0.708 |
| Lung transplant evaluation, n (%) | 6 (20.0%) | 2 (15.4%) | 4 (23.5%) | 0.672 |
| **Psychological history and baseline status** |  |  |  |  |
| Anxiety and/or depression (primary care record)^a^, n (%) | 7 (23.3%) | 3 (23.1%) | 4 (23.5%) | >0.999 |
| Fibromyalgia, n (%) | 3 (10.0%) | 2 (15.4%) | 1 (5.9%) | 0.565 |
| Bereavement, n (%) | 7 (23.3%) | 1 (7.7%) | 6 (35.3%) | 0.104 |
| Predominant concern, n (%) |  |  |  | 0.941 |
| Loss of autonomy | 12 (40.0%) | 5 (38.5%) | 7 (41.2%) |  |
| Disease progression or death | 9 (30.0%) | 3 (23.1%) | 6 (35.3%) |  |
| Secondary losses | 2 (6.7%) | 1 (7.7%) | 1 (5.9%) |  |
| Treatment tolerance | 2 (6.7%) | 1 (7.7%) | 1 (5.9%) |  |
| Other | 5 (16.7%) | 3 (23.1%) | 2 (11.8%) |  |
| Information integration, n (%) |  |  |  | 0.427 |
| Full | 23 (76.7%) | 11 (84.6%) | 12 (70.6%) |  |
| Partial | 7 (23.3%) | 2 (15.4%) | 5 (29.4%) |  |
| Coping style at baseline^b^, n (%) |  |  |  | >0.999 |
| Anxious preoccupation | 9 (30.0%) | 4 (30.8%) | 5 (29.4%) |  |
| Fatalism | 9 (30.0%) | 4 (30.8%) | 5 (29.4%) |  |
| Cognitive avoidance | 6 (20.0%) | 2 (15.4%) | 4 (23.5%) |  |
| Helplessness-hopelessness | 4 (13.3%) | 2 (15.4%) | 2 (11.8%) |  |
| Fighting spirit | 2 (6.7%) | 1 (7.7%) | 1 (5.9%) |  |
| **HADS baseline** |  |  |  |  |
| Anxiety subscale, mean (SD) | 11.5 (4.5) | 12.8 (5.3) | 10.6 (3.8) | 0.179 |
| Anxiety level, n (%) |  |  |  | 0.094 |
| Severe | 10 (33.3%) | 6 (46.2%) | 4 (23.5%) |  |
| Moderate | 7 (23.3%) | 3 (23.1%) | 4 (23.5%) |  |
| Mild | 6 (20.0%) | 0 (0.0%) | 6 (35.3%) |  |
| Normal | 7 (23.3%) | 4 (30.8%) | 3 (17.6%) |  |
| Depression subscale, mean (SD) | 10.9 (4.4) | 12.8 (4.7) | 9.5 (3.7) | 0.093 |
| Depression level, n (%) |  |  |  | 0.051 |
| Severe | 5 (16.7%) | 5 (38.5%) | 0 (0.0%) |  |
| Moderate | 11 (36.7%) | 3 (23.1%) | 8 (47.1%) |  |
| Mild | 7 (23.3%) | 3 (23.1%) | 4 (23.5%) |  |
| Normal | 7 (23.3%) | 2 (15.4%) | 5 (29.4%) |  |
| Data are presented as percentages by column. | | | | |

**Supplementary Table 4.** Baseline characteristics compared between patients with CFS <5 and CFS ≥5. **ILD**: interstitial lung disease. **HP**: hypersensitivity pneumonitis. **CTD-ILD**: connective tissue disease-associated ILD. **IPAF**: interstitial pneumonia with autoimmune features. **FVC**: forced vital capacity (% predicted). **DLCO**: diffusing capacity for carbon monoxide (% predicted). **ILD-GAP**: ILD-Gender, Age, Physiology index. **CFS**: Clinical Frailty Scale. **mMRC**: modified Medical Research Council dyspnoea scale. **HADS**: Hospital Anxiety and Depression Scale. **SD**: standard deviation.

Footnotes: ᵃ Documented in primary care records without formal psychiatric or psychological evaluation; ^b^ Clinically assigned by the treating psychologist; not psychometrically scored (see Methods).

| Characteristics | All patients,  N=30 | CFS <5,  n=16 | CFS ≥5,  n=14 | p-value |
| --- | --- | --- | --- | --- |
| **Sociodemographic characteristics** |  |  |  |  |
| Male, n (%) | 15 (50.0%) | 9 (56.3%) | 6 (42.9%) | 0.464 |
| Age, mean (SD) | 71.0 (8.3) | 70.8 (9.7) | 71.2 (6.8) | 0.886 |
| Civil status, n (%) |  |  |  | 0.511 |
| Married | 22 (73.3%) | 12 (75.0%) | 10 (71.4%) |  |
| Widowed | 6 (20.0%) | 2 (12.5%) | 4 (28.6%) |  |
| Divorced | 1 (3.3%) | 1 (6.3%) | 0 (0.0%) |  |
| Single | 1 (3.3%) | 1 (6.3%) | 0 (0.0%) |  |
| Support network available, n (%) | 29 (96.7%) | 15 (93.8%) | 14 (100.0%) | >0.999 |
| Effective support network, n (%) | 24 (80.0%) | 13 (81.3%) | 11 (78.6%) | >0.999 |
| **Disease and clinical characteristics** |  |  |  |  |
| ILD diagnosis, n (%) |  |  |  | 0.561 |
| Chronic HP | 11 (36.7%) | 5 (31.3%) | 6 (42.9%) |  |
| IPF | 10 (33.3%) | 6 (37.5%) | 4 (28.6%) |  |
| CTD-ILD | 4 (13.3%) | 1 (6.3%) | 3 (21.4%) |  |
| IPAF | 3 (10.0%) | 2 (12.5%) | 1 (7.1%) |  |
| Other | 2 (6.7%) | 2 (12.5%) | 0 (0.0%) |  |
| FVC (% of predicted), mean (SD) | 72.4 (20.9) | 84.1 (18.2) | 59.1 (15.2) | <0.001 |
| DLCO (% of predicted), mean (SD) | 48.7 (15.4) | 56.7 (12.1) | 32.8 (5.6) | <0.001 |
| ILD-GAP index ≥4, n (%) | 17 (56.7%) | 5 (31.3%) | 12 (85.7%) | 0.003 |
| Time since diagnosis in months, mean (SD) | 18.7 (19.2) | 19.4 (23.1) | 18.0 (14.3) | 0.697 |
| ≤6 months, n (%) | 8 (26.7%) | 4 (25.0%) | 4 (28.6%) | >0.999 |
| ≤12 months, n (%) | 14 (46.7%) | 8 (50.0%) | 6 (42.9%) | 0.696 |
| Dyspnoea (mMRC), n (%) |  |  |  | 0.004 |
| Grade 1 | 11 (36.7%) | 8 (50.0%) | 3 (21.4%) |  |
| Grade 2 | 7 (23.3%) | 6 (37.5%) | 1 (7.1%) |  |
| Grade 3 | 12 (40.0%) | 2 (12.5%) | 10 (71.4%) |  |
| Family aggregation, n (%) | 6 (20.0%) | 5 (31.3%) | 1 (7.1%) | 0.175 |
| **Treatment and supportive care** |  |  |  |  |
| Antifibrotic therapy, n (%) | 27 (90.0%) | 15 (93.8%) | 12 (85.7%) | 0.586 |
| Antifibrotic side effects, n (%) | 4 (13.3%) | 4 (25.0%) | 0 (0.0%) | 0.103 |
| O2 on exertion, n (%) | 13 (43.3%) | 2 (12.5%) | 11 (78.6%) | <0.001 |
| O2 at rest, n (%) | 4 (13.3%) | 0 (0.0%) | 4 (28.6%) | 0.037 |
| Opioid use, n (%) | 11 (36.7%) | 2 (12.5%) | 9 (64.3%) | 0.003 |
| Lung transplant evaluation, n (%) | 6 (20.0%) | 1 (6.3%) | 5 (35.7%) | 0.072 |
| **Psychological history and baseline status** |  |  |  |  |
| Anxiety and/or depression (primary care record)^a^, n (%) | 7 (23.3%) | 4 (25.0%) | 3 (21.4%) | >0.999 |
| Fibromyalgia, n (%) | 3 (10.0%) | 2 (12.5%) | 1 (7.1%) | >0.999 |
| Bereavement, n (%) | 7 (23.3%) | 3 (18.8%) | 4 (28.6%) | 0.675 |
| Predominant concern, n (%) |  |  |  | 0.776 |
| Loss of autonomy | 12 (40.0%) | 5 (31.3%) | 7 (50.0%) |  |
| Disease progression or death | 9 (30.0%) | 5 (31.3%) | 4 (28.6%) |  |
| Secondary losses | 2 (6.7%) | 1 (6.3%) | 1 (7.1%) |  |
| Treatment tolerance | 2 (6.7%) | 2 (12.5%) | 0 (0.0%) |  |
| Other | 5 (16.7%) | 3 (18.8%) | 2 (14.3%) |  |
| Information integration, n (%) |  |  |  | 0.204 |
| Full | 23 (76.7%) | 14 (87.5%) | 9 (64.3%) |  |
| Partial | 7 (23.3%) | 2 (12.5%) | 5 (35.7%) |  |
| Coping style at baseline^b^, n (%) |  |  |  | 0.865 |
| Anxious preoccupation | 9 (30.0%) | 5 (31.3%) | 4 (28.6%) |  |
| Fatalism | 9 (30.0%) | 4 (25.0%) | 5 (35.7%) |  |
| Cognitive avoidance | 6 (20.0%) | 3 (18.8%) | 3 (21.4%) |  |
| Helplessness-hopelessness | 4 (13.3%) | 2 (12.5%) | 2 (14.3%) |  |
| Fighting spirit | 2 (6.7%) | 2 (12.5%) | 0 (0.0%) |  |
| **HADS baseline** |  |  |  |  |
| Anxiety subscale, mean (SD) | 11.5 (4.5) | 10.4 (5.0) | 12.9 (3.8) | 0.169 |
| Anxiety level, n (%) |  |  |  | 0.291 |
| Severe | 10 (33.3%) | 4 (25.0%) | 6 (42.9%) |  |
| Moderate | 7 (23.3%) | 3 (18.8%) | 4 (28.6%) |  |
| Mild | 6 (20.0%) | 3 (18.8%) | 3 (21.4%) |  |
| Normal | 7 (23.3%) | 6 (37.5%) | 1 (7.1%) |  |
| Depression subscale, mean (SD) | 10.9 (4.4) | 10.2 (5.0) | 11.7 (3.6) | 0.327 |
| Depression level, n (%) |  |  |  | 0.562 |
| Severe | 5 (16.7%) | 3 (18.8%) | 2 (14.3%) |  |
| Moderate | 11 (36.7%) | 4 (25.0%) | 7 (50.0%) |  |
| Mild | 7 (23.3%) | 4 (25.0%) | 3 (21.4%) |  |
| Normal | 7 (23.3%) | 5 (31.3%) | 2 (14.3%) |  |
| Data are presented as percentages by column. | | | | |
